# eHealth tools for the self-testing of visual acuity: a scoping review

**DOI:** 10.1038/s41746-019-0154-5

**Published:** 2019-08-22

**Authors:** Wai Kent Yeung, Piers Dawes, Annie Pye, Anna-Pavlina Charalambous, Malcolm Neil, Tariq Aslam, Christine Dickinson, Iracema Leroi

**Affiliations:** 1 Division of Neuroscience and Experimental Psychology, University of Manchester and the Manchester Academic Health Sciences Centre, Manchester, UK; 2 Manchester Centre for Audiology and Deafness, University of Manchester and the Manchester Academic Health Sciences, Manchester, UK; 3 Department of Health Sciences, European University Cyprus, Nicosia, Cyprus; 4 University of Dundee, Dundee, UK; 5 Manchester University NHS Foundation Trust, Manchester, UK; 6 Division of Pharmacy & Optometry, University of Manchester and the Manchester Academic Health Sciences Centre, Manchester, UK; 7 Global Brain Health Institute, Trinity College Dublin, Dublin, Ireland

**Keywords:** Refractive errors, Vision disorders, Diagnosis, Visual system, Neuroscience

## Abstract

Uncorrected refractive error is a major cause of vision impairment, and is indexed by visual acuity. Availability of vision assessment is limited in low/middle-income countries and in minority groups in high income countries. eHealth tools offer a solution; two-thirds of the globe own mobile devices. This is a scoping review of the number and quality of tools for self-testing visual acuity. Software applications intended for professional clinical use were excluded. Keyword searches were conducted on Google online, Google Play and iOS store. The first 100 hits in each search were screened against inclusion criteria. After screening, 42 tools were reviewed. Tools assessed near and distance vision. About half (*n* = 20) used bespoke optotypes. The majority (*n* = 25) presented optotypes one by one. Four included a calibration procedure. Only one tool was validated against gold standard measures. Many self-test tools have been published, but lack validation. There is a need for regulation of tools for the self-testing of visual acuity to reduce potential risk or confusion to users.

## Introduction

Vision impairment is rising year on year, with global estimates of 237.1 million affected by moderate or severe vision impairment by 2020.^1^ Visual impairment has negative impacts on quality of life, mental and physical health,^2–6^ impedes performance in school, reduces employability and productivity^7^ and is associated with a greater risk of all-cause mortality in older persons.^8^ Uncorrected refractive errors (URE) and unoperated cataracts are the top global causes of vision impairment and over 80% of all vision impairments are preventable.^9^ UREs are responsible for 53% of vision impairment.^10,11^ URE’s contribution to all moderate-severe vision impairments is estimated to be higher in lower and middle-income countries (LMIC) than in high-income countries e.g.,^11–13^

A potential reason for the prevalence of UREs may be the lack of access to vision care services. In some LMICs, vision care services are only offered at the secondary and tertiary levels of care, not at community level.^7,14^ In high-income countries that do have readily available vision screening, uptake is limited^15^ so UREs may go undetected and uncorrected.^16^ In high-income countries UREs are more common among low socioeconomic and ethnic minority groups.^17–19^ Availability of eye examinations in people’s own homes, living alone, cost of vision care and perceptions that declining visual acuity is normal with ageing are also associated with UREs.^20^ eHealth vision screening tools may help tackle some of the issues relating to visual impairment due to UREs by increasing identification and promoting correction of refractive errors. eHealth tools may also be helpful in identifying visual acuity problems due to other treatable conditions, such as macular degeneration.

Over the past decade, the number of digital health tools has risen. Over 200 online health tools are published every day, and over 318,000 are currently available.^21^ With growth of technology, increasing quantity of online tools, and the potential for savings in healthcare costs, investment in digital health grows year on year.^21^ Two-thirds of the world’s population are now connected via mobile devices.^22^ However, the quality of eHealth tools is uncertain. Developers of eHealth tools generally have no training in healthcare and health professionals are not involved in the development of the majority of tools.^23–25^

Vision assessment is an area of eHealth that may have particularly benefitted from advancements in technology including larger screen sizes and higher screen resolutions, more processing power and lower costs of hardware. Numerous tools for vision assessment have already been developed and published. But the range of tools available has made it difficult for clinicians and the public to determine which tools are the most effective.^26^

The aims of the review were (i) to provide an overview of online or app-based tools for self-testing of visual acuity; and (ii) to identify and critique the quality of these tools with respect to validity and reliability. The review excluded software applications intended for professional clinical use (e.g., the AT20P Acuity Tester^27^ or the Vision Toolbox^28^). The review focussed on tools for self-testing of visual acuity because visual acuity is a strong predictor of self-reported vision related quality of life^29^ and International Classification of Diseases 11 (2018)^30^ definitions of vision impairment are based on visual acuity; visual acuity is the primary index of vision impairment.

## Results

**Fig. 1:**
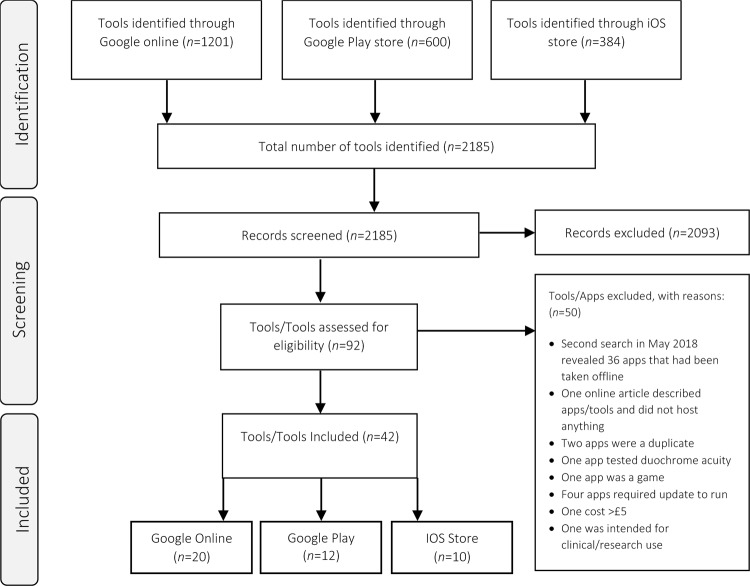
Prisma flow diagram indicating the tools search and screening process

After screening the results, 92 tools were assessed for eligibility, and 42 mobile and/or online tests (Fig. 1) were included for review. All tests took ~5–20 min to complete, including setup and calibration time. Several tests did not specify testing one eye at a time, so were categorised as testing binocular vision. Table 1 provides a description of the tools.

**Table 1:** Description of eHealth visual acuity tools

Test Title	Visual Acuity Type^a^				Optotype^b^						Calibration	Chart Presentation			Platform	Reliability^c^	Validity^c^
	N	F	B	M	SN	SL	E	C	B	O		Singly	Whole	Other			
Online Eye Test	✓	✓		✓			✓				✓	✓			Web		
Eye Chart and Vision Test Online		✓	✓						✓				✓		Web		✓
Online Eye Tests	✓	✓		✓					✓				✓		Web		
Snellen Eye Test Online		✓	✓		✓								✓		Web		
Devlyn Vision Screening	✓	✓		✓					✓		✓	✓			Web		
Snellen Online Eye Test		✓		✓	✓								✓		Web		
Online Eye Test		✓		✓					✓					✓	Web		
Self-Vision Test		✓		✓	✓								✓		Web		
Test Your Eyesight Now		✓		✓	✓								✓		Web		
The Online Eye Test		✓	✓						✓					✓	Web		
Better Vision	✓	✓	✓						✓					✓	Web		
Presbyopia Test	✓			✓					✓				✓		Web		
Vutest	✓			✓					✓					✓	Web	✓	✓
SeeDrivePro		✓		✓		✓					✓			✓	Web	✓	✓
Online Eye Exam		✓		✓					✓				✓		Web		
Online Eye Tests	✓		✓						✓				✓		Web		
Essilor Vision Test	✓			✓			✓					✓			Web		
Online Eye Exam		✓		✓	✓								✓		Web		
Online Eye Tests	✓			✓			✓					✓			Web		
Zeiss Online Vision Screening	✓		✓				✓				✓	✓			Web		
EyeTester		✓	✓					✓				✓			App		
EyeTesterPro		✓	✓					✓				✓			App		
REST Rapid Eye Screening Test		✓	✓				✓					✓			App	✓	✓
Eye Test by Boots Opticians	✓			✓					✓			✓			App		
Advanced VISION Test	✓		✓				✓		✓			✓			App		
Eye Test Free	✓			✓					✓			✓			App		
Eyesight Checking	✓		✓				✓	✓				✓			App		
OPSM Eye Check	✓	✓		✓					✓			✓			App		
Vision Scan Lite	✓			✓					✓			✓			App		
Vision Scan Universal	✓			✓					✓			✓			App		
Eye Checker	✓	✓	✓						✓			✓			App		
Eye Doctor Trainer - Vision Up	✓		✓						✓					✓	App		
Eye Exam - Andrew Brusentsov	✓			✓			✓	✓	✓	✓		✓			App		
Eye Exam Pro - Andrew Brusentsov	✓			✓			✓	✓	✓	✓		✓			App		
Eye Exercises - Eye Care Plus	✓			✓			✓	✓	✓	✓		✓			App		
Eye Test Charts	✓	✓	✓					✓	✓				✓		App		
Eye Test Girls	✓		✓				✓					✓			App		
Eye Vision Test	✓			✓						✓		✓			App		
EyeXam	✓	✓		✓				✓				✓			App		
iKit - For Your Eyes Only	✓	✓		✓					✓			✓			App		
OPSM Eye Check	✓	✓		✓					✓			✓			App		
Vision Test	✓	✓		✓					✓			✓			App		

^a^*N* Near Vision; *F* Far Vision; *B* Binocular; *M* Monocular;

^b^*SN* Snellen; *SL* Sloan; *E* Tumbling E; *C* Landolt C; *B* Bespoke; *O* Others

^c^Grey shading reported as having data, but not provided, or in preparation

### Optotypes

There were twenty-one tools using a mixture of standard optotypes; these included Sloan Letters, original Snellen, tumbling E and Landolt C. Within these twenty-one tools, five also offered non-standard optotypes and three included options for pictures or numbers. Twenty tools provided alternative optotypes only; these included an assortment of serif and sans serif typefaces. One tool included numerical optotypes only.

### Presentation and response interface

The majority of tools (*n* = 25) presented optotypes one by one and none provided optotypes with crowding as an option. This format was employed for optotypes that required the participant to decide on the orientation of the figure, for example Landolt C or tumbling E. For mobile devices, a swiping gesture could be used to record response to the direction of the optotype. The second most common presentation format was to present the entire chart (*n* = 11). Ten were found through Google Online searches and were intended for use with a desktop/laptop computer interface. There was also a small number that presented the optotypes using non-validated methods, for example line by line (e.g., better vision by Cambridge Institute for Better Vision; SeeDrivePro by EyeLab Ltd), or within a circle of selectable letters (e.g., vutest by EyeLab Ltd).

### Calibration

The majority of tools (*n* = 38) provided instructions for completing the test in a standard way, for example, positioning at the correct distance from the display. Six tools (e.g., Eye Chart and Vision Test Online by MindBluff.com; Online Eye Tests by Ross Brown Optometry; The Online Eye Test by Jim Allen; vutest by EyeLab Ltd; Online Eye Tests by Dr Oliver Findl and Online Eye Tests, Free eye exams and vision test by Prokerala;) provided information on the testing distance dependent on the resolution of the display. Three additional tools (e.g., Snellen Online Eye Test by Smart Buy Glasses; Self- Vision test by Milford Eye Care; Online Eye Exam by Jem Optical) included a standard Snellen chart with the addition of a calibration bar. The calibration bar must be measured in centimetres by the user and the result is the distance that the user should stand from the display in feet. One tool (Snellen Eye Test Online by eyes-and-vision.com) asked the user to measure the largest optotype (i.e., the optotype that sits at the top of the vision chart) in inches. This result is then multiplied by a constant to obtain the distance the user must be from the display.

Four tools provided a calibration procedure, which involved standardising the optotype size by using a reference object (such as a credit card) to adjust the image sizes appropriately using on screen toggles (e.g., Online Eye Test by Easee Online; Devlyn Vision Screening by Devlyn Optical; SeeDrivePro by EyeLab Ltd; Zeiss Online Vision Screening by Zeiss). Calibration of one tool (Online Eye Test by Easee Online) involved taking into account screen brightness, screen placement (i.e., monitor/laptop position), and colour calibration. The tools that offered calibration procedures were only those on web-hosted platforms. No tools on mobile platforms provided calibration options.

The majority of tools (*n* = 32) included a statement that they were screening tools and were not intended as a replacement for professional testing. The tools recommended consulting a professional if the user had any concerns about their vision, and recommended regular vision testing.

### Presentation of results

The presentation of results varied widely. The most common (*n* = 13) was the Snellen fraction with the majority measured in feet (*n* = 9).^31^ Five of those thirteen tools simply provided information on the scoring of the chart and the user would then be required to interpret the results by following the instructions. For example, the user would be required to determine Snellen score based on which line was read (e.g., the third line would be equivalent to 20/40). The remaining 11 tools automatically provided a Snellen fraction along with an explanation (e.g., “20/100 means that when you stand 20 feet from the chart you can see what a normal person standing 100 feet away can see, 20/100 is considered moderate vision impairment”).

Eighteen tools provided a visual performance score but without any further interpretation. For example, some tools provide a percentage score or visual performance categorised as low, medium or high risk. The calculation of risk score was not explained, nor what the “risk” score related to (e.g., OPSM Eye Check by OPSM; Devlyn Vision Screening by Devlyn Vision) Two tools (Vutest and SeeDrivePro by EyeLab Ltd) required a monetary payment in order to retrieve the results.

Four tools produced a simple decimal value with no further clarifications. These decimal scores may be derived from Snellen scores, however this was not detailed (Eye Tester and Eye Tester Pro by Fuso Precision; Eye Checker by Nanny_786; Eye Doctor Trainer—Vision Up by BytePioneers S.R.O.). Two tools provided a dioptre-based prescription for near vision; Nanyang optical recommended the lens strength for the user, and the test by Easee Online provided prescriptions with values relating to sphere, cylinder and vision percentage per eye.

Lastly, five tools (Eye Test Free by Magostech Information System Pvt Ltd; Eye Exam and Eye Exam Pro by Andrew Brusentsov; Eye Exercises—Eye Care Plus by healthcare4mobile; Vision Test by NeoVize Group) gave a non-standard percentage-based score for each eye (e.g., L80%, R75%) with no details of how these percentages are calculated or interpreted.

The Easee Online test also provided a prescription service at a cost to the user. The prescription generated by the online test was validated by the in-house specialist at Easee Online and an eyewear prescription dispatched to the user.

### Validity and reliability

**Fig. 2:**
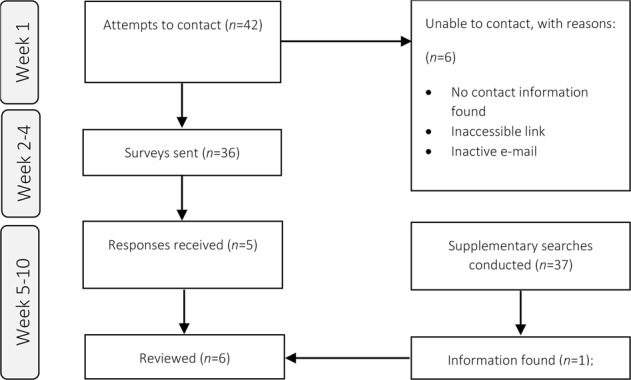
Prisma flow diagram indicating attempts to contact and survey the tool developers for further information

Five electronic surveys were returned, and a further 37 supplementary searches were conducted to uncover validity or reliability data (Fig. 2). Survey responses were received for the “Online Eye Test” by Easee, the “Eye Chart and Vision Test” by mindbluff.com, “Vutest” and “Seedrive Pro” by EyeLab Ltd and the “Eye Test” by Boots Opticians (Table 1). Supplementary searches identified information for three additional tools, however only one of these yielded relevant data (REST by Zu Quan Ik).

The five tools that responded to the survey reported the collection of reliability and validity data, but did not present any data to support this being the case.

Jan-Bond et al. conducted a study to determine the agreement and intraclass correlation coefficient (ICC) of visual acuity between the REST rapid eye screening test *(Zu Quan Ik)* and an Early Treatment Diabetic Retinopathy Study (ETDRS) tumbling E chart.^32^ The REST utilises a tumbling E chart, with calibrated optotype sizes to 1 and 3-m testing distances. Visual acuity was tested at 3-m distance on both the ETDRS and the REST with 101 adult participants who were patients and staff (mean age *=* 37.0 ± 15.9; range 5.0–75.0) of the eye clinic at Hospital Universiti Sains Malaysia. Data were not provided on the eye conditions of participants. Participants with acuity worse than 6/60 were excluded. The ethnic background of participants reflected the Malaysian general population, with the majority being Malays (62.4%). However, the sample was not representative in terms of educational background; over half of the participants were professionals with tertiary level education. Strong agreements were observed between the two tools for the right (ICC = 0.91; 95%CI from 0.86 to 0.94, *p* < 0.001) and left (ICC = 0.93; 95%CI from 0.90 to 0.95, *p* < 0.001) eye. The REST was also significantly quicker to administer than ETDRS. A second study compared REST with ETRDS chart among 200 adult patients with immature cataracts. This study also reported similar relationships between REST and ETRDS results (Pearson’s *r* *=* 0.99, *p* *<* 0.001).^33^ Reliability of the REST was not ascertained to our knowledge.

## Discussion

Online vision self-assessment tools may help address the increasing burden of visual impairment in both high-income countries and in LMIC. But of the 42 tools for self-assessment of visual acuity that were included for review, only one tool reported validity data. And for that one tool, the number and types of patients in the validation were not reported. No tools reported reliability data. The uncertain validity and reliability of most tools is a concern because unreliable tools may miss cases of visual impairment, or may cause undue anxiety by falsely identifying cases. Poor quality eHealth apps may compromise patient safety (e.g., in relation to dose calculation,^34^ or melanoma detection^35^). There may be similar issues for vision assessment tools. The vision assessment tools that were identified in the mobile app stores tended to be categorised under “Medical” or “Health and Fitness” sections. Use of such terminology may increase the likelihood of users relying on these tools for medical information. There is a need to establish regulatory standards for vision self-assessment tools to ensure that user safety is not compromised.

A variety of models of regulation are available. National governmental regulatory authorities (e.g., the US Food and Drug Administration or the UK Medicines and Healthcare products Regulatory Agency [MHRA]) may struggle to keep up with the rapid development of eHealth tools.^36^ Voluntary certification or the European Union’s system of decentralised registration are alternatives to regulation by central national authorities.^37^ For example, a voluntary certification system was developed based on a set of standards developed by commercial eHealth companies.^38^ The system involved app developers paying to have their apps certified as meeting the prescribed standards. In the European Union’s model, app developers can file an application for medical device registration with any member state of the European Union.^39^ The Conformité Européenne (CE) mark that is issued by the respective body in each member state is then valid throughout the European Union.

However, these registration systems all have limitations. For example, the FDA only regulates apps that are defined by the FDA as being a medical device, or apps that may risk patient safety, and most apps fall outside of these definitions.^40^

The voluntary industry-led registration system mentioned above^38^ was discontinued after security flaws were identified in some certified apps and after low uptake of registration by app developers. The main limitation of the European Union’s system of certification is that registration standards only relate to standards of safety and performance but do not require clinical efficacy of the device to be established.^37^

One potential solution may be endorsement by credible national health agencies and/or third sector organisations based on an internationally agreed set of standards that take into account the efficacy and safety of the eHealth tool. The United Kingdom’s National Health Service (NHS) recently created an online register of digital health tools for self-care of various health conditions that meet standards of quality, safety and effectiveness (https://apps.beta.nhs.uk/). Standards are assessed based on digital assessment questions (DAQ), a framework developed by subject matter experts.^41^ Healthcare organisations elsewhere could establish similar registries of eHealth apps according to DAQ standards.

Another potential solution is based on the Clinical Laboratory Improvement Amendments of 1988 (CLIA) model.^42^ CLIA is a system for ensuring that diagnostic testing laboratories comply with US regulatory standards. Non-profit accrediting agencies with authority to issue certification under US federal CLIA standards ensure consistency of record keeping and staff training.^43^ The CLIA model could be applied to eHealth tools to ensure that they comply with basic standards of accessibility (the inclusion of clear language, ease of use, affordability and usability), privacy and security (assurances that the tool appropriately secures private health data, and compliance with data sharing laws [e.g., general data protection regulation]) and content (apps are developed with healthcare professional involvement, with accurate information and limited monetisation practices).^37,44^ As US healthcare providers do not do business with laboratories that are not CLIA certified, users could choose to use eHealth tools with appropriate certification.

Many professional clinical assessment systems utilise technology similar to those used by the tools covered in this review (e.g., Thomson Software Solutions’ products^28^). Professional systems reliably assess a much wider range of parameters than visual acuity, the typical focus of self-test apps. Developers of most self-test apps may not have a background in vision healthcare.^23–25^ It may therefore be that any lack of reliability in self-testing apps is not a technological problem, but is due to lack of awareness on the part of app developers concerning the optometrically important features of the test (e.g., the style of the optotypes or their spacing). Guidelines that involve a minimum quality standards may be useful in ensuring that self-test apps are of comparable reliability to professional systems.

In addition to systems of quality certification for eHealth tools, eHealth tools for vision impairment should include a clear link to care that supports users acting on the result of the vision test. The visual acuity self-assessment tools in this review directed the user to visit a professional for further advice or testing. Not including a specific link to clinical or support services may mean that only a small proportion of people who fail a vision self-test act on the results. Additional possibilities may include directly linking self-test apps with clinical services, and/or remotely delivered care via video conferencing, or other technologies. This type of telehealth care is on the rise, for example in the remote delivery of a cardiac rehabilitation exercise programme^45^ or in the self-management of skincare,^46^ where an eHealth app enables two-way communication between patient and clinician. The Easee Online tool identified in this review conducts an eye exam and provides the user with a prescription, which can then be issued (for a fee) by Easee optometrists. The remote care paradigm presented by Easee is of interest, but a serious limitation of this paradigm is the use of an internet tool of uncertain reliability that cannot provide a complete examination of ocular health and which may result in users being falsely reassured by the finding of normal visual acuity.

Utilising video-conferencing and telehealth methods may facilitate the reach of clinical services in underserved areas (such as in LMICs). A study conducted on an Indian population looked at the efficacy of a computerised visual acuity screening system, and report good agreement between the computerised telehealth method versus face-to-face assessment.^47^

A limitation of this review was that it only offers a snapshot of self-assessment vision tools at one point in time in a rapidly changing online landscape. However the issues related to quality and links to care identified in this review are likely to continue to pertain to eHealth self-assessment vision tools that may be released in the months and years following this review. A further limitation is that only tools for self-testing of visual acuity were evaluated; tools may assess additional parameters that may relate to specific eye health conditions. However, the focus on visual acuity is valid, as acuity is one of the primary indices of vision impairment.

## Conclusion

eHealth vision tools have potential to meet a growing burden associated with vision impairment, particularly in LMIC. But the validity and reliability of most has not been established. There is a need to ensure that vision eHealth vision tools are of good quality. Solutions include following established frameworks for regulation that take into account accessibility, privacy and content, and the creation of repositories of high quality tools by national health agencies or third sector organisations that users can depend on. There is also a need for effective linking self-tests of visual acuity with a care pathway, which could also involve remotely delivered care.

## Methods

### Design

A systematic search was conducted between May 2017 and May 2018 to identify candidate screening tools for inclusion. The search engine Google was used to identify online tools and Google Play and the Apple App Store were used to identify smartphone and tablet applications. The search terms used were “Online” OR “Internet-based” AND “Vision” OR “Eye” AND “Test” OR “Screen” OR “Check”. For the online tools search, the additional search term “Online” was included in combination with the other search terms. On both Google Play and Google online searches, the first hundred hits were screened. These were sorted automatically according to relevance by the search engine. Location services were disabled, and adverts were not included. For the iOS store, all results returned were screened, as each search combination often returned fewer than hundred results.

A first reviewer screened all the titles, identifying candidate tools for inclusion. A second reviewer then screened 10% of the titles to ensure consensus opinion. All candidate tests for inclusion were screened by both reviewers and in instances of disagreement, a third reviewer decided on whether the measure met criteria for inclusion.

Inclusion criteria were (i) tests were intended as self-administered tests of visual acuity that can be completed without the support of a professional; (ii) tests provide some feedback on performance; (iii) tests were hosted online or as a smartphone application; (iv) tests were freely available or low cost; an upper limit of £5 was chosen to select tools that are readily available and within the average price range for mobile apps.^48^ Tests designed for use by professionals were excluded.

### Survey design

A survey was devised to obtain information about the design, availability, development and validity of the tools. The survey was adapted from a survey of clinical cognitive assessments developed by Snyder et al. with permission from the author^49^ (see Supplementary Table 1). Survey data were collected via an internet-based questionnaire over a 3-month period. A contact email was identified for each tool (where available), and an email was sent to the developer that contained a link to the online survey, explained the purpose of the survey and invited the developer to respond. Developers were sent periodic reminder emails at fortnightly intervals until a response was received or until 10 weeks had passed. For tools for which no response was received, or no contact could be identified, supplementary searches were conducted. Searches were conducted on Google (to identify grey literature), Google Scholar and Medline (to identify published/peer reviewed articles). The search terms used were “(name of developer, if available)” AND “(name of the tool)”. The first hundred results (sorted by relevance) were used. Applicable titles and webpages were reviewed for their relevance, and relevant materials were downloaded and saved. Data were extracted from these materials according to a data extraction form based on questions asked in the survey.

### Regulatory approvals

None of the tools were registered with a health regulatory authority that we were aware of. The Easee tool was reported to be certified as a CE class 1 Medical device, which is the lowest risk category of medical devices. Although self-certified CE marking is an indicator of safety and performance, it does not certify clinical efficacy.

## Supplementary information

Supplementary Table 1

Supplementary Data

## Data Availability

The authors declare that the data supporting the findings of this study are available within the paper and its supplementary information files.

## Supplementary information

**Supplementary Information** accompanies the paper on the *npj Digital Medicine* website (10.1038/s41746-019-0154-5).
