# Author Correction: eHealth tools for the self-testing of visual acuity: a scoping review

**DOI:** 10.1038/s41746-019-0195-9

**Published:** 2019-11-26

**Authors:** Wai Kent Yeung, Piers Dawes, Annie Pye, Anna-Pavlina Charalambous, Malcolm Neil, Tariq Aslam, Christine Dickinson, Iracema Leroi

**Affiliations:** 1 Division of Neuroscience and Experimental Psychology, University of Manchester and the Manchester Academic Health Sciences Centre, Manchester, UK; 2 Manchester Centre for Audiology and Deafness, University of Manchester and the Manchester Academic Health Sciences, Manchester, UK; 3 Department of Health Sciences, European University Cyprus, Nicosia, Cyprus; 4 University of Dundee, Dundee, UK; 5 Manchester University NHS Foundation Trust, Manchester, UK; 6 Division of Pharmacy & Optometry, University of Manchester and the Manchester Academic Health Sciences Centre, Manchester, UK; 7 Global Brain Health Institute, Trinity College Dublin, Dublin, Ireland

**Correction to:** *npj Digital Medicine* 10.1038/s41746-019-0154-5, published online 22 August 2019

In the original version of the published Article, Anna-Pavlina Charalambous was mistakenly omitted from the Author list and Author Contributions statement. Anna-Pavlina Charalambous has been added as the 4th Author, and is affiliated with the Department of Health Sciences, European University Cyprus, Nicosia, Cyprus. The Author Contributions statement has been updated as follows: W.K.Y. conducted an update of the searches and wrote the manuscript including the introduction, methods, results and discussion. P.D. and A.P. wrote the review protocol including the search strategy and methodology, and had significant input in writing the introduction, methods, results and discussion sections. A.P.C., A.P., and M.N. conducted the initial searches. T.A. and C.D. contributed to the development of the manuscript and acted as expert consultants on the ophthalmological aspects of the review. I.L. and P.D. are principal investigators for the SENSE-Cog project, and oversaw the development of the review. This has been corrected in the HTML and PDF version of the Article.

