# Supplementary material for: eHealth tools for the self-testing of visual acuity: a scoping review
Source: NPJ Digit Med. 2019 Aug 22;2:82. doi: 10.1038/s41746-019-0154-5 (PMC6706420; doi:10.1038/s41746-019-0154-5)
Supplement: Supplementary file 1 — Supplementary Table 1 [file 41746_2019_154_MOESM1_ESM.pdf]

Supplementary Table 1: Survey items sent to developers of eHealth tools

Section	Topic	Question(s)	
**Section 1**	General description of measure and measure development	Please enter a link or URL for your tool.	
		Name of the tool	
		Is your test available	Online<br>On an Android device<br>On an Apple device<br>Other (Please specify)
		Please provide a brief description of your test	
		What visual functions does your test measure?	Distance Visual Acuity<br>Near Visual Acuity<br>Low Contrast Visual Acuity<br>Contrast Sensitivity<br>Central Visual Field<br>Binocular Vision<br>Refractive Correction<br>Colour Vision<br>Vision-Related Quality of Life<br>Symptom Questionnaire
		Does your test include assessment of 'red flag' symptoms that could indicate serious visual pathology?	Yes<br>No<br>If yes, please provide details
		Is your test in the public domain/proprietary?	
		If proprietary, please describe how to obtain access to the instrument.	
**Section 2**	Normative data	Have normative data been collected for your test?	Yes<br>No

			If yes, please provide details of normative data (populations, relevant publications, tables, documents)
**Section 3**	Reliability, validity and responsiveness	Which, if any, of the following validity, reliability and responsiveness analyses have been undertaken with your test?	Sensitivity for detecting change over time<br>Convergence with similar tests<br>Convergence with tests of functional ability<br>Predictive validity<br>Sensitivity and/or specificity<br>Construct and face validity<br>Test-retest reliability<br>Internal consistency
		Please provide details of any analyses and relevant publications.	
		Please describe the kind of feedback that the user receives upon completing the measure.	
**Section 4**	Data collection and storage	Can your test be completed by lay people without support from trained technicians or clinicians?	Yes<br>No
		What technology and hardware requirements are there for running your test?	
		How long does it take to complete your test in minutes?	
		How are test results displayed, stored and transmitted?	
		Are test results accessible in a real-time (or close to real-time manner?	Yes<br>No
		Does your data collection and storage system allow for the provision of real-time alerts regarding significant vision change, for use by the user?	Yes<br>No

		If yes, who would receive that alert?	The User<br>Caregivers or family members<br>The supervising clinician<br>Other (please specify)
**Section 5**	Quality assurance and approvals	Does your technology allow for any automation of quality assurance/ quality checking of data inputted by the user?	Yes<br>No<br>If yes, please describe
		Has your test been accepted by any health regulatory authorities (e.g. national regulatory authority, European Medicines Agency (EMA), US Food and Drug Administration (FDA)) as a valid measure for any particular disease entity?	Yes<br>No<br>If yes, please describe.
**Section 6**	Availability and accessibility	Are there any physical, cognitive or psychological restrictions that might limit test completion?	
		Are there any culturally or linguistically adapted test versions?	Yes<br>No<br>If yes, please describe.
**Section 7**	Other information	If you have any other comments about your tool that we did not cover in this survey, please list them below.	
		Please provide an email address that we might use to contact you should we have further questions regarding your tool.	
